# Serum testosterone, sex hormone‐binding globulin and sex‐specific risk of incident type 2 diabetes in a retrospective primary care cohort

**DOI:** 10.1111/cen.13862

**Published:** 2018-10-23

**Authors:** Michael W. O’Reilly, Marija Glisic, Balachandran Kumarendran, Anuradhaa Subramanian, Konstantinos N. Manolopoulos, Abd A. Tahrani, Deepi Keerthy, Taulant Muka, Konstantinos A. Toulis, Wasim Hanif, G. Neil Thomas, Oscar H. Franco, Wiebke Arlt, Krishnarajah Nirantharakumar

**Affiliations:** ^1^ Institute of Metabolism and Systems Research University of Birmingham Birmingham UK; ^2^ Centre for Endocrinology, Diabetes and Metabolism Birmingham Health Partners Birmingham UK; ^3^ Department of Epidemiology Erasmus University Medical Centre Rotterdam The Netherlands; ^4^ Institute of Applied Health Research University of Birmingham Birmingham UK; ^5^ Department of Public Health, Faculty of Medicine University of Kelaniya Kelaniya Sri Lanka; ^6^ Institute of Social and Preventive Medicine (ISPM) University of Bern Bern Switzerland

**Keywords:** androgens, diabetes, hypogonadism, metabolic diseases, population health, sex hormone‐binding globulin, testosterone

## Abstract

**Objective:**

Previous studies suggest that androgens have a sexually dimorphic impact on metabolic dysfunction. However, the sex‐specific link between circulating androgens and risk of type 2 diabetes mellitus (T2DM) has not been examined in a large scale, longitudinal cohort, a task we undertook in this study.

**Design:**

A retrospective cohort study in a UK primary care database.

**Patients:**

We included men and women with available serum testosterone and sex hormone‐binding globulin (SHBG) results.

**Measurements:**

We categorized serum concentrations according to clinically relevant cut‐off points and calculated crude and adjusted T2DM Incidence Rate Ratios (IRRs and aIRRs).

**Results:**

Serum testosterone concentrations were available in 70 541 men and 81 889 women; serum SHBG was available in 15 907 men and 42 034 women. In comparison to a reference cohort with serum testosterone ≥20 nmol/L, men with lower serum testosterone had a significantly increased risk of T2DM, with the highest risk in those with serum testosterone <7 nmol/L (aIRR 2.71, 95% CI 2.34‐3.14, *P* < 0.001). In women, the risk of T2DM started to increase significantly when serum testosterone concentrations exceeded 1.5 nmol/L, with the highest risk in women with serum testosterone ≥3.5 nmol/L (aIRR 1.98, 95% CI 1.55‐2.52, *P* < 0.001). These observations were verified in a continuous rather than categorized analysis. The risk of T2DM increased in men and women with serum SHBG <40 and <50 nmol/L, respectively.

**Conclusions/Interpretation:**

In this longitudinal study, we found sexually dimorphic associations between serum testosterone and risk of incident T2DM. Androgen deficiency and excess should be considered important risk factors for diabetes in men and women, respectively.

## 1 INTRODUCTION

Sex differences are critical in the epidemiology and pathophysiology of metabolic disease, with an increased incidence of type 2 diabetes mellitus (T2DM) and cardiovascular disease in men.^1^ Sex hormones such as androgens may mediate these differences, but the association between androgens and metabolic dysfunction is complex and sex‐specific.^2^ Androgen excess has recently been identified as an independent risk factor for non‐alcoholic fatty liver disease (NAFLD) in women^3^ and promotes lipid accumulation in female adipose tissue as well as systemic lipotoxicity.^4^ Female‐to‐male gender reassignment patients undergoing androgen therapy develop dyslipidemia and abnormal body composition.^5^, ^6^ Mirroring this, the adverse metabolic phenotype of male androgen deficiency bears a striking similarity to that of female androgen excess; lower testosterone levels in men are associated with impaired glucose homoeostasis, hepatic steatosis and coronary artery disease.^1^, ^7^, ^8^ A number of meta‐analyses support a sex‐specific relationship between androgens and the risk of metabolic dysfunction and suggest that low circulating sex hormone‐binding globulin (SHBG) concentrations may be metabolically harmful in both sexes.^8^, ^9^

Delineating an independent role for androgens in the pathogenesis of T2DM is confounded by changes in body composition, body mass index and lean mass observed in disorders of androgen excess and deficiency.^10^ Against the background of a global epidemic of T2DM,^11^ there is an urgent health need to understand the sexually dimorphic role played by androgens in the pathogenesis of hyperglycaemia. The shared constellation of risk factors observed in women with androgen excess and men with androgen deficiency suggests that circulating androgen concentrations common to both disorders may be metabolically disadvantageous.^2^ To our knowledge, however, no large longitudinal studies have specifically examined the association between circulating androgen exposure per se and risk of T2DM in a sex‐specific context.

The aim of this study was to investigate the independent sex‐specific association between serum testosterone concentrations and the risk of hyperglycaemia in men and women by undertaking a retrospective cohort study in a large and diverse UK population base.

## 2 MATERIALS AND METHODS

### 2.1 Database

A large primary care database in the UK with contribution from over 700 general practices (14 million patients) was utilized for this study. Data from practices that use VISION Electronic Medical Record (EMR) are gathered, anonymized and released for research purpose.^12^ The resulting database, The Health Improvement Network (THIN) database holds data on demographic characteristics, clinical diagnosis, physical measurement, laboratory results and prescriptions. The THIN database is broadly representative of the UK population structure^13^ and has been utilized for numerous epidemiological studies, including studies on T2DM^14^^,^ 15 and sex hormones.^3^, ^14^, ^15^

### 2.2 Testosterone and sex hormone‐binding globulin (SHBG) measurements

Men or women over the age of 16 who had a measurement of the serum concentration of testosterone or SHBG between 1st of January 2000 and 15th of May 2016 were eligible to take part in the study. Common clinical indications for these measurements include suspected polycystic ovary syndrome (PCOS) in women, infertility investigations in both sexes and erectile dysfunction in men.^16^, ^17^ Where multiple measurements were available in one individual, the first measurement was utilized. Patients with the outcome of interest (T2DM) preceding the index date were excluded from the study.

### 2.3 Exposure categories

To explore non‐linear relationships, establish gradient increase and assess risk within the normal range, measurements were categorized by applying clinically relevant cut‐off points for serum concentrations (nmol/L).^3^ For women, testosterone was grouped as <1.0 nmol/L (reference group), 1.0‐1.49, 1.5‐1.99, 2.0‐2.49, 2.5‐2.99, 3.0‐3.49 and >3.5 nmol/L. For men, the groups were as follows: <7, 7‐9.9, 10.0‐14.9, 15‐19.9, >20.0 nmol/L (reference group) nmol/L. For both sexes, SHBG was categorized as >60.0 nmol/L (reference group), 50.0‐59.9, 40.0‐49.9, 30.0‐39.9, 20.0‐29.9 and <20 nmol/L. Exposures were also treated as continuous variables and risk of T2DM assessed.

### 2.4 Follow‐up period

The date of measurement of testosterone or SHBG served as the index date. Each participant was followed up from the index date until the exit date. Exit date was defined as the earliest of the following dates: outcome (diagnosis of T2DM), study end, death or the date they left the general practice or the general practice stopped contributing to the database.

### 2.5 Outcome and covariates

A clinical diagnosis of T2DM by the general practitioner was the outcome of interest. In the UK, the Quality Outcome Framework (QOF) in general practices ensures high‐quality data on important comorbidities such as cardiovascular disease, hypertension and T2DM.^18^ Within the database, diagnostic codes for T2DM were identified based on Read codes, a hierarchical coding system to record signs, symptoms, procedures and diagnosis in primary care.^3^ Covariates that are independent predictors of T2DM other than the exposure of interest were selected on the basis of biological plausibility and previous literature.^19^ These included age, body mass index (BMI), Townsend deprivation score and smoking status.

### 2.6 Statistical analysis

Baseline data of each category in the serum testosterone and SHBG cohorts were reported separately for men and women as mean (standard deviation) or median (interquartile range [IQR]) as appropriate for continuous variables and as proportions for categorical variables. Crude Incidence Rate Ratio (IRR) and adjusted Incidence Rate Ratio (aIRR) were calculated by applying Poisson regression offsetting for the person‐years of follow‐up. Covariates adjusted for in the model were age, BMI, Townsend quintiles and smoking status. In women, an additional model included polycystic ovary syndrome (PCOS) as a covariate to explore if the risk of T2DM in women was independent of a diagnosis of PCOS. In an additional sensitivity analysis, when adjusting for PCOS. We accepted the presence of hirsutism and anovulation as indicative of PCOS given that the diagnosis is underreported in primary care.

Where missing data existed (BMI, Townsend or smoking), we created a separate category so that all available data is utilized in the analysis. BMI was categorized as per WHO recommendation into <25.0, 25‐29.0 and >30 kg/m^2^. All analyses were performed in Stata 14.0 (StataCorp LLC, College Station, TX, USA).

### 2.7 Subgroup analysis

In women, we performed stratified analysis by age (<50 and 50 years and above) to explore if the association was similar before and after the average age of menopause. A similar age‐stratified analysis was also carried out in men. In addition to this, in those patients with simultaneous measurements of testosterone and SHBG, a free androgen index (FAI) was calculated ([T × 100]/SHBG), and risk of T2DM calculated to control for the confounding effect of low SHBG levels.

### 2.8 Ethical approval

This study used routinely collected, anonymized primary care data. Patients were not involved in the study, and therefore, no consent was required. Research using THIN data was approved by the NHS South‐East Multicentre Research Ethics Committee in 2003, with the condition that studies undergo independent scientific review.^20^ Approval for this analysis was obtained from the Scientific Review Committee for the use of THIN data in January 2018 (SRC reference number 17THIN106).

## 3 RESULTS

### 3.1 Characteristics of the cohorts with serum testosterone and SHBG measurements

A total of 152 430 participants in the cohort with available serum testosterone measurement results (testosterone cohort; 70 541 men and 81 889 women) and a total of 57 941 participants (15 907 men and 42 034 women) in the SHBG cohort, both derived from the THIN database, met the inclusion criteria and were included in the current study. Median follow‐up in the testosterone cohort was 3.3 years (IQR:1.5‐6.1) in men and 3.2 (IQR:1.3‐6.2) years in women. In the SHBG cohort, median follow‐up was 2.8 (1.3‐4.9) years in men and 2.8 (1.2‐5.4) in women. The mean age for men was 51.6 (SD 14.8) years in the testosterone cohort and 51.7 (SD 16.0) years in the SHBG cohort. For women, mean age was 33.2 (SD 10.9) years in the testosterone cohort and 32.1 (SD 10.6) years in the SHBG cohort. In total, 40 464 (57.4%) men in the testosterone cohort and 9795 (61.6%) men in the SHBG cohort were overweight or obese (BMI ≥ 25 kg/m^2^). Among women, 36 640 (44.7%) were obese or overweight in the testosterone cohort and 19 270 (45.8%) in the SHBG cohort. Approximately 21% of men and 22% of women were smokers across both testosterone and SHBG cohorts (Table 1). A diagnosis of PCOS was only documented in 6.3% (N = 5136) and 7.9% (N = 3303) of the female testosterone and SHBG cohorts, respectively. However, clinical features suggestive of PCOS, anovulation and clinical evidence of hirsutism, were documented in 25.8% and 11.2% of the female testosterone cohort, respectively, and in 26.9% and 12.1% of the female SHBG cohort, respectively.

**Table 1:** Baseline characteristics of the testosterone and SHBG cohorts stratified by sex

Characteristics	Men		Women	
	Serum testosterone	Serum SHBG	Serum testosterone	Serum SHBG
Population, n (%)	70 541 (46.28)	15 907 (27.45)	81 889 (53.72)	42 034 (72.55)
Age (years), mean (SD)	51.6 (14.8)	51.7 (16.0)	33.2 (10.9)	32.1 (10.6)
Townsend index n (%)				
1 (least deprived)	20 017 (28.38)	3997 (25.13)	18 470 (22.55)	8753 (20.82)
2	15 481 (21.95)	3427 (21.54)	15 688 (19.16)	7688 (18.29)
3	13 687 (19.40)	3033 (19.07)	17 043 (20.81)	8681 (20.65)
4	10 997 (15.59)	2565 (16.12)	15 295 (18.68)	8155 (19.40)
5 (most deprived)	7374 (10.45)	2186 (13.74)	10 269 (12.54)	5955 (14.17)
Missing or implausible data	2985 (4.23)	699 (4.39)	5124 (6.26)	2802 (6.67)
BMI (kg/m^2^) categorized, n (%)				
<25	19 195 (27.21)	3995 (25.11)	32 519 (39.71)	15 975 (38.00)
25‐30	25 962 (36.80)	5817 (36.57)	16 849 (20.58)	8445 (20.09)
>30	14 502 (20.56)	3978 (25.01)	19 791 (24.17)	10 825 (25.75)
Missing or implausible data	10 882 (15.43)	2117 (13.31)	12 730 (15.55)	6789 (16.15)
Smoking status, n (%)				
Non‐smokers	53 311 (75.57)	12 264 (77.10)	61 288 (74.84)	31 557 (75.07)
Smokers	15 325 (21.72)	3377 (21.23)	18 020 (22.01)	9312 (22.15)
Missing or implausible data	1905 (2.70)	266 (1.67)	2581 (3.15)	1165 (2.77)
Confounding conditions				
PCOS			5136 (6.27)	3303 (7.86)
Anovulation			21 148 (25.83)	11 288 (26.85)
Hirsutism			9133 (11.15)	5064 (12.05)
Follow‐up in years, median (IQR)	3.3 (1.5‐6.1)	2.8 (1.3 ‐ 4.9)	3.2 (1.3‐6.2)	2.8 (1.2‐5.4)

Biochemical evidence of male androgen deficiency (serum testosterone < 7 nmol/L) was observed in 5862 men (8.3%). Biochemical evidence of female androgen excess (serum testosterone > 2 nmol/L) was observed in 20 565 women (25.1%); of those, 2481 women (3.0%) had severe androgen excess (serum testosterone ≥ 3.5 nmol/L). Serum SHBG concentrations < 20 nmol/L were observed in 2517 (15.8%) men and 3733 (8.9%) women (Supporting Information Tables S1‐S4).

### 3.2 Association between sex hormones and T2DM risk in men

Among 70 541 men with serum testosterone measurements, 3156 developed T2DM during the follow‐up period. As expected, increasing age, overweight/obesity, smoking and higher social deprivation conferred an increased risk for T2DM (Supporting Information Tables S5 and S6).

**Figure 1:**
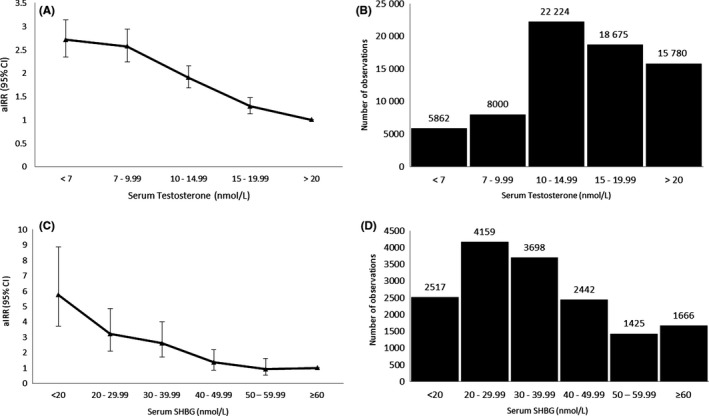
Risk of incident type 2 diabetes (T2DM) according to serum testosterone and sex hormone‐binding globulin (SHBG) concentration categories in men. A, Adjusted Incidence Rate Ratios (aIRRs) for diabetes in 70 541 men with serum testosterone measurements. B, Distribution of 70 541 men across each quintile of serum testosterone concentration. C, aIRRs for serum SHBG concentrations for incident diabetes in 15 907 men. D, Distribution of 15 907 men across each category of serum SHBG concentration. [Correction added on 14 November 2018, after first online publication: in panel D, an error in the data for 40‐49.99 nmol/L has been corrected.]

After adjusting for age, BMI, Townsend index and smoking status, aIRR for T2DM in men increased with decreasing categories of serum testosterone concentrations, most notably a 271% higher risk of developing T2DM in those with testosterone levels < 7 nmol/L, compared to the reference category of ≥20 nmol/L (aIRR 2.71, 95% CI 2.34‐3.14, *P* < 0.001, Table 2). However, the risk of T2DM increased even within the normal male testosterone range (15‐19.99 nmol/L, aIRR 1.29, 95% CI 1.13‐1.47, *P* < 0.001; 10‐14.99 nmol/L, aIRR 1.90, 95% CI 1.68‐2.15, *P* < 0.001, Table 2 & Figure 1A,B).

**Table 2:** Risk of incident T2DM according to the category of serum testosterone and SHBG at baseline

	IRR (95% CI); *P*‐value				
	Adjusted^a^	Adjusted^b^	Adjusted^c^	Adjusted^d^	
Men					
Serum testosterone concentration categories (nmol/L)					
<7	3.82 (3.31‐4.41); *P* < 0.001	2.60 (2.25‐3.00); *P* < 0.001	2.71 (2.34‐3.14); *P* < 0.001		
7‐9.99	3.70 (3.24‐4.22); *P* < 0.001	2.46 (2.15‐2.81); *P* < 0.001	2.57 (2.24‐2.94); *P* < 0.001		
10‐14.99	2.40 (2.13‐2.71); *P* < 0.001	1.83 (1.62‐2.06); *P* < 0.001	1.90 (1.68‐2.15); *P* < 0.001		
15‐19.99	1.45 (1.27‐1.66); *P* < 0.001	1.25 (1.09‐1.43); *P* = 0.001	1.29 (1.13‐1.47); *P* < 0.001		
≥20	*Ref*	*Ref*	*Ref*		
Serum SHBG concentration categories (nmol/L)					
<20	8.23 (5.37‐12.63); *P* < 0.001	5.00 (3.24‐7.71); *P* < 0.001	5.74 (3.72‐8.87); *P* < 0.001		
20‐29.99	4.30 (2.83‐6.53); *P* < 0.001	2.92 (1.91‐4.44); *P* < 0.001	3.20 (2.09‐4.87); *P* < 0.001		
30‐39.99	3.33 (2.19‐5.08); *P* < 0.001	2.45 (1.60‐3.74); *P* < 0.001	2.61 (1.71‐3.99); *P* < 0.001		
40‐49.99	1.56 (0.98‐2.50); *P* = 0.063	1.28 (0.80‐2.06); *P* = 0.298	1.36 (0.85‐2.17); *P* = 0.207		
50‐59.99	1.07 (0.61‐1.87); *P* = 0.825	0.88 (0.50‐1.54); *P* = 0.654	0.91 (0.52‐1.60); *P* = 0.748		
≥60	*Ref*	*Ref*	*Ref*		
Women					
Serum testosterone concentration categories (nmol/L)					
<1	*Ref*	*Ref*	*Ref*		*Ref*
1.0‐1.49	1.21 (1.02‐1.43); *P* = 0.030	1.12 (0.95‐1.33); *P* = 0.184	1.12 (0.94‐1.32); *P* = 0.204		1.11 (0.94‐1.32); *P* = 0.213
1.5‐1.99	1.45 (1.23‐1.70); *P* < 0.001	1.26 (1.07‐1.48); *P* = 0.005	1.23 (1.05‐1.45); *P* = 0.011		1.23 (1.04‐1.44); *P* = 0.013
2.0‐2.49	1.70 (1.42‐2.04); *P* < 0.001	1.34 (1.12‐1.61); *P* = 0.002	1.30 (1.08‐1.56); *P* = 0.005		1.28 (1.07‐1.54); *P* = 0.008
2.5‐2.99	2.07 (1.67‐2.58); *P* < 0.001	1.59 (1.27‐1.97); *P* < 0.001	1.53 (1.23‐1.90); *P* < 0.001		1.50 (1.20‐1.87); *P* < 0.001
3.0‐3.49	2.51 (1.90‐3.32); *P* < 0.001	1.74 (1.31‐2.30); *P* < 0.001	1.68 (1.27‐2.23); *P* < 0.001		1.62 (1.22‐2.15); *P* = 0.001
≥3.5	3.00 (2.36‐3.82); *P* < 0.001	2.09 (1.64‐2.67); *P* < 0.001	1.98 (1.55‐2.52); *P* < 0.001		1.89 (1.48‐2.42); *P* < 0.001
Serum SHBG concentration categories (nmol/L)					
<20	19.76 (14.36‐27.21); *P* < 0.001	8.96 (6.42‐12.50); *P* < 0.001	9.23 (6.61‐12.88); *P* < 0.001	9.13 (6.53‐12.75); *P* < 0.001	
20‐29.99	8.66 (6.29‐11.93); *P* < 0.001	4.45 (3.20‐6.19); *P* < 0.001	4.48 (3.22‐6.24); *P* < 0.001	4.44 (3.19‐6.18); *P* < 0.001	
30‐39.99	4.66 (3.31‐6.57); *P* < 0.001	2.69 (1.90‐3.82); *P* < 0.001	2.70 (1.91‐3.84); *P* < 0.001	2.69 (1.90‐3.82); *P* < 0.001	
40‐49.99	2.99 (2.04‐4.38); *P* < 0.001	2.05 (1.40‐3.02); *P* < 0.001	2.08 (1.41‐3.05); *P* < 0.001	2.07 (1.41‐3.05); *P* < 0.001	
50‐59.99	1.64 (1.02‐2.64); *P* = 0.043	1.29 (0.80‐2.08); *P* = 0.295	1.29 (0.80‐2.07); *P* = 0.304	1.29 (0.80‐2.08); *P* = 0.301	
≥60	*Ref*	*Ref*	*Ref*	*Ref*	

IRR, incidence rate ratio; SHBG, sex hormone‐binding globulin; T2DM, type 2 diabetes mellitus

a Adjusted for age.

b Adjusted for age, BMI.

c Adjusted for age, BMI, Townsend index, smoking status.

d Adjusted for age, BMI, Townsend index, smoking status, PCOS.

In the SHBG cohort, among 15 907 men studied, there were 708 cases of incident T2DM during the follow‐up period. After adjusting for age, BMI, Townsend index and smoking status, the risk of T2DM increased in men with SHBG levels < 40 nmol/L; aIRR of incident T2DM increased across categories of decreasing SHBG concentrations as compared to the reference category (≥60 nmol/L) and the risk was more than 5‐fold higher in the group with SHBG < 20 nmol/L (aIRR 5.74, 95% CI 3.72‐8.87, Table 2 & Figure 1C,D).

### 3.3 Association between sex hormones and T2D risk in women

**Figure 2:**
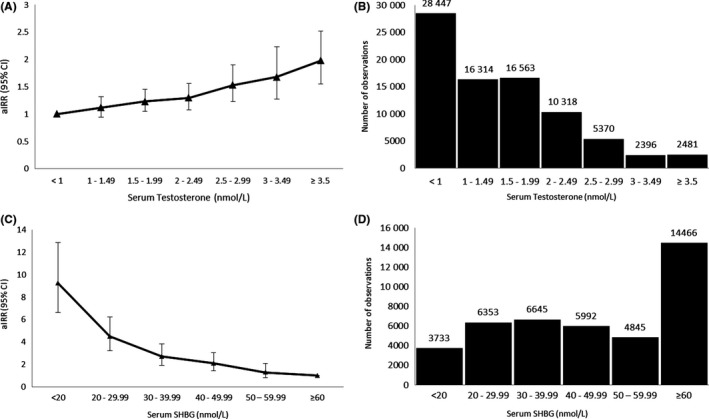
Risk of incident type 2 diabetes (T2DM) according to serum testosterone and sex hormone‐binding globulin (SHBG) concentrations in women. A, Adjusted Incidence Rate Ratios (aIRRs) for incident diabetes in 81 889 women with serum testosterone measurements. B, Distribution of 81 889 women across each category of serum testosterone concentration. C, aIRRs for serum SHBG concentrations for incident diabetes in 42 034 women with serum SHBG measurements. D, Distribution of 42 034 women across each category of serum SHBG concentration. [Correction added on 14 November 2018, after first online publication: in panel B, missing data for 3–3.49 and ≥3.5 nmol/L have been added.]

Among 81 889 women with serum testosterone measurements, 1282 developed T2DM during the follow‐up period. After adjusting for age, BMI, Townsend index and smoking status, T2DM aIRR tended to be higher with increasing serum testosterone levels. The risk increased significantly for serum testosterone levels > 1.5 nmol/L, as compared to reference category (<1 nmol/L), and continued to increase across each category of serum testosterone concentrations thereafter, with a twofold increase in risk observed in women with serum testosterone ≥ 3.5 nmol/L (aIRR 1.98, 95% CI 1.55‐2.52, *P* < 0.001, Table 2 & Figure 2A,B). Further adjustment for a diagnosis of PCOS or clinical features of suspected PCOS (hirsutism or anovulation) did not substantially change results (aIRR in subgroup of women with testosterone levels > 3.5 nmol/L = 1.89, 95% CI 1.48‐2.42, *P* < 0.001 and 1.76, 95% CI 1.38‐2.25, *P* < 0.001 respectively, Supporting Information Table S7).

In the SHBG cohort, among 42 034 women studied, there were 597 cases of incident T2DM during the follow‐up period. The risk of incident T2DM increased with each category of decreasing SHBG concentration. Women with serum SHBG concentrations <20 nmol/L had a 9‐fold higher risk of developing T2DM compared to the reference category of ≥60 nmol/L (aIRR 9.23, 95% CI 6.61‐12.88, *P* < 0.001), after adjustment for age, BMI, Townsend index and smoking status (Table 2 & Figure 2C,D). Additional adjustment for a diagnosis of PCOS and clinical features of suspected PCOS did not alter the risk of T2DM (aIRR 9.13, 95% CI 6.53‐12.75, *P* < 0.001 and aIRR 8.88, 95% CI 6.36‐12.42, *P* < 0.001, respectively, Table S8).

### 3.4 Analysis of sex hormones as a continuous variable

In men, for every nmol/L decrease in testosterone, the risk of T2DM increased by 5% (aIRR 1.05, 95% CI 1.04‐1.06, *P* < 0.001). In women, for every nmol/L increase in testosterone, the risk of T2DM increased by 10% (aIRR 1.10, 95% CI 1.06‐1.14, *P* < 0.001). In the analysis of SHBG, for every nmol/L decrease in SHBG the risk of T2DM increased by 3% in both men and women (aIRR 1.03, 95% CI 1.03‐1.04, *P* < 0.001, in both sexes).

### 3.5 Free androgen index and risk of T2DM

Only 40% women (n = 34 578) and 16% of men (n = 12 178) had undergone a simultaneous measurement of serum SHBG and testosterone. Using these to calculate the free androgen index (FAI), we found that FAI was positively associated with risk of T2DM in women (aIRR 1.03, 95% CI 1.02‐1.04, *P* < 0.001), but not in men (aIRR 1.00, 95% CI 0.997‐1.004, *P* = 0.789).

### 3.6 Subgroup analyses

Subgroup analysis stratified by age (<50 and ≥50 years) did not alter the observed associations. In both age groups, a gradient increase in risk of T2DM was observed with increasing testosterone concentrations in women and decreasing testosterone concentrations in men (Figure S1; Tables S9‐S12). Increased aIRRs for T2DM were noted with lower concentrations of SHBG in both age groups in men and women (Figure S2; Tables S13‐S16).

## 4 DISCUSSION

In this large retrospective cohort study, we have demonstrated that androgens confer an independent sex‐specific effect on the risk of incident T2DM. To our knowledge, this is the largest study, and the first longitudinal analysis, to address the impact of serum testosterone on risk of development of T2DM in both men and women. In the female cohort, aIRRs for T2DM increased significantly once serum testosterone concentrations increased above 1.5 nmo/L; even those with circulating testosterone levels between 1.5 and 1.99 nmol/L, conventionally considered within the normal physiological range for women, already had a 23% increased risk of T2DM compared to the reference group. Perhaps even more surprisingly, once male serum testosterone concentrations dropped below 20 nmol/L, the risk of T2DM began to increase; men with circulating concentrations between 15 and 19.99 nmol/L, that is within the normal physiological male range, had a 28% increased risk of T2DM over the study period. Reduced SHBG concentrations in both sexes, but particularly in women, also potently increased the risk of T2DM. This finding is in agreement with observations from some previous studies, which demonstrated a stronger inverse association between SHBG levels and risk of T2DM in women compared to men.^9^, ^21^ This inverse relationship with T2DM appears to be particularly strong in postmenopausal women.^22^ A 2011 meta‐analysis, however, found that higher SHBG levels were equally associated with a reduced risk of metabolic syndrome in both sexes.^23^

A systematic review and meta‐analysis, which included a total of 3825 men and 4795 women in 36 cross‐sectional studies, as well as 368 cases from 7 prospective study populations, previously demonstrated that increased serum testosterone was associated with a 60% higher risk of T2DM in women; higher testosterone levels in men reduced the risk of T2DM by 42%.^9^ Goodman‐Gruen *et al* 24 also observed sex differences in the association between serum androgens and glucose tolerance status in an older community cohort of 775 men and 633 women above the age of 55. Men with impaired fasting glucose, impaired glucose tolerance and T2DM had significantly lower levels of serum testosterone, while women with T2DM had significantly higher levels of bioavailable testosterone, independent of total body fat, fat distribution, physical activity and smoking. However, our study is the only longitudinal retrospective analysis to comprehensively evaluate these associations.

A number of key insights into the role of androgen excess in the development of metabolic dysfunction are provided by studies in women with polycystic ovary syndrome (PCOS), a disorder affecting up to 10% of the female population and primarily defined by the presence of hyperandrogenism and ovulatory dysfunction.^25^ We have recently demonstrated that lean women with PCOS have an almost twofold increased risk of NAFLD, a hepatic manifestation of metabolic dysfunction, and that androgen excess is an independent mediator of this increased risk.^3^ Androgen‐mediated adipose tissue lipotoxicity may contribute to this increase in NAFLD risk.^4^, ^26^ PCOS women are at significantly increased risk of impaired glucose tolerance and T2DM at a young age, irrespective of body weight.^27^ A recent large Danish population register study concluded that the risk of T2DM was fourfold higher for women with PCOS, and diagnosed 4 years earlier, compared to women in the background population.^28^

Male androgen deficiency occurs as a consequence of primary testicular pathology, hypothalamic‐pituitary disorders, obesity or as part of the ageing process in older men.^29^, ^30^ Additionally, iatrogenic hypogonadism due to androgen deprivation therapy is observed in men with prostate cancer.^31^ Whilst the relationship between obesity and hypogonadism in men is complex and bidirectional,^32^ data from male cohorts treated with short‐term androgen deprivation therapy show that hypogonadism directly induces metabolically deleterious changes in body composition, with increases in weight and in percentage fat body mass.^33^ However, studies of androgen deprivation therapy, which result in significant hypogonadism, are not an ideal model to compare to the relatively modest reductions in testosterone observed in community‐dwelling older men. The results of our study are particularly surprising, given that an increased risk of T2DM was apparent at circulating testosterone concentrations considered physiologically normal, but below the reference group of 20 nmol/L, independent of age, obesity and other potential confounding factors. However, our results do not imply endorsement of testosterone pharmacotherapy to restore circulating testosterone levels above 20 nmol/L in otherwise healthy men. Studies investigating a potential beneficial impact of androgen therapy on metabolic outcomes in men with testosterone concentrations in the low or low‐normal range have shown at best conflicting results. A recent double‐blind placebo‐controlled trial of testosterone treatment in 788 older men showed no impact on serum glucose or HbA1C^34^; another study showed no change in insulin sensitivity after 36 months of treatment in 308 community‐dwelling men.^35^ The 2018 Endocrine Society Clinical Practice Guideline on testosterone therapy in men with hypogonadism no longer recommend screening men with T2DM for low serum testosterone, and advise against using testosterone therapy to improve glycaemic control.^36^

Low circulating SHBG has been consistently identified as a surrogate marker for T2DM in both sexes in a number of smaller studies and meta‐analyses,^9^, ^37^, ^38^ and our study supports these observations. In a meta‐analysis of 13 population‐based studies with 1912 incident cases of T2DM, low SHBG was associated with increased risk of T2DM in women, irrespective of menopausal status.^37^ SHBG levels are typically higher in women, and our data confirm that reduced circulating concentrations are associated with a higher risk of T2DM than that observed in men. SHBG is a critical mediator of the association between sex steroids and metabolic dysfunction. The majority of circulating testosterone is bound to SHBG, such that only the unbound or “free” fraction is capable of exerting effects in target tissues.^39^ Therefore, reduced SHBG levels in women are a surrogate marker of increased circulating active androgens. Insulin is a potent regulator of hepatic SHBG output, which is suppressed in the context of hyperinsulinaemia, leading to reduced SHBG, and therefore increased free androgens, in insulin‐resistant states such as PCOS in women.^40^ It is unlikely, however, that SHBG independently plays a causal role in the pathophysiology of metabolic diseases such as T2DM. Low SHBG and testosterone levels in men are likely to be mediated by obesity in a population already at increased risk.^41^ We found that FAI in men did not have a negative linear association with T2DM risk, indicating that low SHBG rather than testosterone is the predominantly associated with metabolic risk in men. This supports the observations of Bhasin,^42^ but conflicts with those of Haring *et al*,^43^ who found that declining testosterone rather than SHBG levels were the main driver of metabolic syndrome in a large German cohort. It is important to note that FAI must be interpreted with caution in both men and women, and is particularly inaccurate in women when the SHBG concentration falls below 30 nmol/L.^44^

This study has a number of important limitations, not least its retrospective nature. Detailed clinical phenotyping in studies of this type is not possible. There are also no detailed data available on laboratory assays used to measure serum testosterone. This is not of particular concern in men, as physiologically higher testosterone concentrations do not represent a challenge for quantification by either radioimmunoassay (RIA) or tandem mass spectrometry techniques. In women, however, where low circulating concentrations pose significant analytical and quantification difficulties for standard RIAs, the consensus is that today measurements should be performed by liquid chromatography‐tandem mass spectrometry to improve quantification and avoid cross‐reactivity.^45^ Furthermore, we have no information on the time of day blood sampling for serum testosterone took place; in men, Endocrine Society guidelines emphasize that morning samples are crucial to accurately diagnose hypogonadism.^46^ Lastly, we must assume that testosterone data were obtained from men and women with a clinical indication for serum measurement; this introduces a potential bias by indication. However, we believe that these limitations are ameliorated by the large patient numbers and the clear and potent gradient towards sex‐specific T2DM risk in the study population.

In conclusion, in the largest retrospective longitudinal study of its kind, we have demonstrated evidence of a sexually dimorphic role for androgens in mediating the risk of T2DM. Reduced SHBG levels in both sexes, but particularly in women, significantly increase the risk of T2DM. These data further define androgens as a novel metabolic risk factor in men and women, but potential mechanisms underpinning these observations remain to be clarified. We suggest that women with androgen excess and men with androgen deficiency should be systematically screened for T2DM. Future studies will be required to show if reducing androgens in women, and increasing androgens in men, will improve overall metabolic health and risk of progression to overt T2DM.

## CONFLICT OF INTEREST

The authors have no conflict of interest to declare.

## AUTHOR CONTRIBUTIONS

MWOR, WA and KN conceptualized the manuscript. MG, BK, AS, KAT and KN designed the methodology. MG, KM, AS and KN performed data cleaning and analysis. MWOR, MG, BK, AS, WA, KNM and KN wrote the manuscript. MWOR, BK, AS, TM, WH, KAT, KNM, AAT OHF, KN and WA reviewed and edited the final manuscript. WA and KN were responsible for overall supervision. All authors contributed to the interpretation of the data and approved the final manuscript for submission.
