# Supplementary material for: Serum testosterone, sex hormone‐binding globulin and sex‐specific risk of incident type 2 diabetes in a retrospective primary care cohort
Source: Clin Endocrinol (Oxf). 2018 Oct 23;90(1):145–54. doi: 10.1111/cen.13862 (PMC6334272; doi:10.1111/cen.13862)
Supplement: Supplementary file 1 [file CEN-90-145-s001.docx]

**Supplementary Table 1: Characteristics of men with available measurement result for serum testosterone (N=70,541).**

| **MEN**  **(N=70,541)** | **Serum testosterone concentration categories (nmol/L)** | | | | |
| --- | --- | --- | --- | --- | --- |
|  | **< 7** | **7 - 9.99** | **10 - 14.99** | **15 - 19.99** | **> 20** |
| **Population, n (%)** | 5,862 (8.31) | 8,000 (11.34) | 22,224 (31.50) | 18,675 (26.47) | 15,780 (22.37) |
| **Age, mean (SD)** | 56.61 (16.89) | 53.43 (13.79) | 52.62 (13.88) | 50.90 (14.38) | 48.41 (15.30) |
| **Townsend index n (%)** |  |  |  |  |  |
| ***1 (least deprived)*** | 1719 (29.32) | 2200 (27.50) | 6534 (29.40) | 5434 (29.10) | 4130 (26.17) |
| ***2*** | 1272 (21.70) | 1813 (22.66) | 5022 (22.60) | 4073 (21.81) | 3301 (20.92) |
| ***3*** | 1153 (19.67) | 1621 (20.26) | 4202 (18.91) | 3633 (19.45) | 3078 (19.51) |
| ***4*** | 884 (15.08) | 1241 (15.51) | 3357 (15.11) | 2889 (15.47) | 2626 (16.64) |
| ***5 (most deprived)*** | 593 (10.12) | 827 (10.34) | 2138 (9.62) | 1881 (10.07) | 1935 (12.26) |
| ***Missing or implausible data*** | 241 (4.11) | 298 (3.72) | 971 (4.37) | 765 (4.10) | 710 (4.50) |
| **BMI (kg/m^2^) categorised, n (%)** |  |  |  |  |  |
| ***<25*** | 1211 (20.66) | 1363 (17.04) | 5066 (22.80) | 5500 (29.45) | 6055 (38.37) |
| ***25-30*** | 2093 (35.70) | 2982 (37.28) | 8856 (39.85) | 7082 (37.92) | 4949 (31.36) |
| ***>30*** | 1876 (32.00) | 2710 (33.88) | 5281 (23.76) | 3072 (16.45) | 1563 (9.90) |
| ***Missing or implausible data*** | 682 (11.63) | 945 (11.81) | 3021 (13.59) | 3021 (16.18) | 3213 (20.36) |
| **Smoking status, n (%)** |  |  |  |  |  |
| ***Non-smokers*** | 4818 (82.19) | 6554 (81.92) | 17580 (79.10) | 13972 (74.82) | 10387 (65.82) |
| ***Smokers*** | 911 (15.54) | 1270 (15.88) | 4152 (18.68) | 4203 (22.51) | 4789 (30.35) |
| ***Missing or implausible data*** | 133 (2.27) | 176 (2.20) | 492 (2.21) | 500 (2.68) | 604 (3.83) |

**Supplementary Table 2: Characteristics of men with available measurement result for serum sex-hormone binding globulin (SHBG) (N=15,907).**

| **MEN**  **(N=15,907)** | **Serum SHBG concentration categories (nmol/L)** | | | | | |
| --- | --- | --- | --- | --- | --- | --- |
|  | **<20** | **20 - 29.99** | **30 - 39.99** | **40 - 49.99** | **50 – 59.99** | **≥60** |
| **Population, n (%)** | 2517 (15.82) | 4159 (26.15) | 3698 (23.25) | 2442 (15.35) | 1425 (8.96) | 1666 (10.47) |
| **Age, mean (SD)** | 43.20 (12.98) | 48.80 (14.04) | 52. 55 (13.94) | 55.03 (13.86) | 57.63 (13.47) | 60.21 (14.10) |
| **Townsend index n (%)** |  |  |  |  |  |  |
| ***1 (least deprived)*** | 609 (24.20) | 1044 (25.1) | 979 (26.47) | 612 (25.06) | 361 (25.33) | 392 (23.53) |
| ***2*** | 531 (21.10) | 906 (21.78) | 774 (20.93) | 537 (21.99) | 314 (22.04) | 365 (21.91) |
| ***3*** | 494 (19.63) | 802 (19.28) | 722 (19.52) | 472 (19.33) | 251 (17.61) | 292 (17.53) |
| ***4*** | 415 (16.49) | 655 (15.75) | 580 (15.68) | 391 (16.01) | 228 (16) | 296 (17.77) |
| ***5 (most deprived)*** | 335 (13.31) | 569 (13.68) | 497 (13.44) | 317 (12.98) | 207 (14.53) | 261 (15.67) |
| ***Missing or implausible data*** | 133 (5.28) | 183 (4.40) | 146 (3.95) | 113 (4.63) | 64 (4.49) | 60 (3.60) |
| **BMI (kg/m^2^) categorised, n (%)** |  |  |  |  |  |  |
| ***<25*** | 377 (14.98) | 813 (19.55) | 863 (23.34) | 722 (29.57) | 478 (33.54) | 742 (44.54) |
| ***25-30*** | 860 (34.17) | 1482 (35.63) | 1466 (39.64) | 942 (38.57) | 537 (37.68) | 530 (31.81) |
| ***>30*** | 864 (34.33) | 1229 (29.55) | 897 (24.26) | 492 (20.15) | 272 (19.09) | 224 (13.45) |
| ***Missing or implausible data*** | 416 (16.53) | 635 (15.27) | 472 (12.76) | 286 (11.71) | 138 (9.68) | 170 (10.20) |
| **Smoking status, n (%)** |  |  |  |  |  |  |
| ***Non-smokers*** | 2039 (81.01) | 3307 (79.51) | 2861 (77.37) | 1875 (76.78) | 1048 (73.54) | 1134 (68.07) |
| ***Smokers*** | 410 (16.29) | 770 (18.51) | 776 (20.98) | 539 (22.07) | 366 (25.68) | 516 (30.97) |
| ***Missing or implausible data*** | 68 (2.70) | 82 (1.97) | 61 (1.65) | 28 (1.15) | 11 (0.77) | 16 (0.96) |

**Supplementary Table 3: Characteristics of women with available measurement result for serum testosterone (N=81,889).**

| **WOMEN**  **(N=81,889)** | **Serum testosterone concentration categories (nmol/L)** | | | | | | |
| --- | --- | --- | --- | --- | --- | --- | --- |
|  | **< 1** | **1 - 1.49** | **1.5 - 1.99** | **2 - 2.49** | **2.5 - 2.99** | **3 - 3.49** | **≥ 3.5** |
| **Population, n (%)** | 28,447 (34.74) | 16,314 (19.92) | 16,563 (20.23) | 10,318 (12.60) | 5,370 (6.56) | 2,396 (2.93) | 2,481 (3.03) |
| **Age, mean (SD)** | 37.06 (11.42) | 33.29 (10.36) | 31.59 (9.79) | 29.91 (9.27) | 28.47 (8.87) | 27.95 (8.94) | 28.87 (11.23) |
| **Townsend index n (%)** |  |  |  |  |  |  |  |
| ***1 (least deprived)*** | 6903 (24.27) | 3748 (22.97) | 3679 (22.21) | 2161 (20.94) | 1060 (19.74) | 466 (19.45) | 453 (18.26) |
| ***2*** | 5664 (19.91) | 3091 (18.95) | 3171 (19.15) | 1941 (18.81) | 961 (17.90) | 433 (18.07) | 427 (17.21) |
| ***3*** | 5906 (20.76) | 3414 (20.93) | 3396 (20.50) | 2161 (20.94) | 1137 (21.17) | 527 (21.99) | 502 (20.23) |
| ***4*** | 4948 (17.39) | 3026 (18.55) | 3155 (19.05) | 2025 (19.63) | 1102 (20.52) | 494 (20.62) | 545 (21.97) |
| ***5 (most deprived)*** | 3188 (11.21) | 2036 (12.48) | 2141 (12.93) | 1351 (13.09) | 799 (14.88) | 348 (14.52) | 406 (16.36) |
| ***Missing or implausible data*** | 1838 (6.46) | 999 (6.12) | 1021 (6.16) | 679 (6.58) | 311 (5.79) | 128 (5.34) | 148 (5.97) |
| **BMI (kg/m^2^) categorised, n (%)** |  |  |  |  |  |  |  |
| ***<25*** | 13135 (46.17) | 6563 (40.23) | 6157 (37.17) | 3587 (34.76) | 1683 (31.34) | 695 (29.01) | 699 (28.17) |
| ***25-30*** | 6159 (21.65) | 3407 (20.88) | 3396 (20.50) | 1906 (18.47) | 1028 (19.14) | 462 (19.28) | 491 (19.79) |
| ***>30*** | 5721 (20.11) | 3795 (23.26) | 4206 (25.39) | 2935 (28.45) | 1564 (29.12) | 757 (31.59) | 813 (32.77) |
| ***Missing or implausible data*** | 3432 (12.06) | 2549 (15.62) | 2804 (16.93) | 1890 (18.32) | 1095 (20.39) | 482 (20.12) | 478 (19.27) |
| **Smoking status, n (%)** |  |  |  |  |  |  |  |
| ***Non-smokers*** | 23225 (81.64) | 12421 (76.14) | 11838 (71.47) | 7079 (68.61) | 3607 (67.17) | 1569 (65.48) | 1549 (62.43) |
| ***Smokers*** | 4713 (16.57) | 3406 (20.88) | 4128 (24.92) | 2780 (26.94) | 1491 (27.77) | 702 (29.30) | 800 (32.25) |
| ***Missing or implausible data*** | 509 (1.79) | 487 (2.99) | 597 (3.60) | 459 (4.45) | 272 (5.07) | 125 (5.22) | 132 (5.32) |
| **Medical Condition, n (%)** |  |  |  |  |  |  |  |
| ***PCOS*** | 737 (2.59) | 749 (4.59) | 1077 (6.50) | 973 (9.43) | 680 (12.66) | 445 (18.57) | 475 (19.15) |
| ***Anovulation*** | 5885 (20.69) | 3782 (23.18) | 4391 (26.51) | 3163 (30.66) | 1941 (36.15) | 961 (40.11) | 1025 (41.31) |
| ***Hirsutism*** | 2055 (7.22) | 1682 (10.31) | 2045 (12.35) | 1514 (14.67) | 915 (17.04) | 404 (16.86) | 518 (20.88) |
| ***PCOS/Anovulation/Hirsutism*** | 7960 (27.98) | 5537 (33.94) | 6513 (39.32) | 4743 (45.97) | 2863 (53.31) | 1390 (58.01) | 1503 (60.58) |

**Supplementary Table 4: Characteristics of women with available measurement result for serum sex-hormone binding globulin (SHBG) (N=42,034).**

| **WOMEN**  **(N=42,034)** | **Serum SHBG concentration categories (nmol/L)** | | | | | |
| --- | --- | --- | --- | --- | --- | --- |
|  | **<20** | **20 - 29.99** | **30 - 39.99** | **40 - 49.99** | **50 - 59.99** | **≥60** |
| **Population, n (%)** | 3733 (8.88) | 6353 (15.11) | 6645 (15.81) | 5,992(14.25) | 4845 (11.53) | 14,466(34.41) |
| **Age, mean (SD)** | 28.00 (9.53) | 30 .31 (10.41) | 31.65 (10.67) | 32.01 (10.37) | 32.89 (10.42) | 33.95 (10.62) |
| **Townsend index n (%)** |  |  |  |  |  |  |
| ***1 (least deprived)*** | 688 (18.43) | 1187 (18.68) | 1324 (19.92) | 1281 (21.38) | 1058 (21.84) | 3215 (22.22) |
| ***2*** | 587 (15.72) | 1061 (16.70) | 1221 (18.37) | 1115 (18.61) | 858 (17.71) | 2846 (19.67) |
| ***3*** | 769 (20.6) | 1309 (20.60) | 1353 (20.36) | 1229 (20.51) | 1032 (21.3) | 2989 (20.66) |
| ***4*** | 814 (21.81) | 1340 (21.09) | 1341 (20.18) | 1127 (18.81) | 914 (18.86) | 2619 (18.10) |
| ***5 (most deprived)*** | 648 (17.36) | 1057 (16.64) | 988 (14.87) | 861 (14.37) | 655 (13.52) | 1746 (12.07) |
| ***Missing or implausible data*** | 227 (6.08) | 399 (6.28) | 418 (6.29) | 379 (6.33) | 328 (6.77) | 1051 (7.27) |
| **BMI (kg/m^2^) categorised, n (%)** |  |  |  |  |  |  |
| ***<25*** | 400 (10.72) | 1084 (17.06) | 1736 (26.12) | 2253 (37.60) | 2201 (45.43) | 8301 (57.38) |
| ***25-30*** | 601 (16.10) | 1291 (20.32) | 1505 (22.65) | 1382 (23.06) | 1073 (22.15) | 2593 (17.92) |
| ***>30*** | 2014 (53.95) | 2858 (44.99) | 2278 (34.28) | 1380 (23.03) | 804 (16.59) | 1491 (10.31) |
| ***Missing or implausible data*** | 718 (19.23) | 1120 (17.63) | 1126 (16.95) | 977 (16.31) | 767 (15.83) | 2081 (14.39) |
| **Smoking status, n (%)** |  |  |  |  |  |  |
| ***Non-smokers*** | 2747 (73.59) | 4662 (73.38) | 4882 (73.47) | 4449 (74.25) | 3636 (75.05) | 11181 (77.29) |
| ***Smokers*** | 809 (21.67) | 1469 (23.12) | 1543 (23.22) | 1382 (23.06) | 1088 (22.46) | 3021 (20.88) |
| ***Missing or implausible data*** | 177 (4.74) | 222 (3.49) | 220 (3.31) | 161 (2.69) | 121 (2.50) | 264 (1.82) |
| **Medical Condition, n (%)** |  |  |  |  |  |  |
| ***PCOS*** | 668 (17.89) | 872 (13.73) | 560 (8.43) | 393 (6.56) | 231 (4.77) | 579 (4.00) |
| ***Anovulation*** | 1303 (34.90) | 1972 (31.04) | 1857 (27.95) | 1627 (27.15) | 1206 (24.89) | 3323 (22.97) |
| ***Hirsutism*** | 685 (18.35) | 1087 (17.11) | 916 (13.78) | 764 (12.75) | 499 (10.30) | 1113 (7.69) |
| ***PCOS/Anovulation/Hirsutism*** | 2074 (55.56) | 3159 (49.72) | 2780 (41.84) | 2383 (39.77) | 1706 (35.29) | 4545 (31.42) |

**Supplementary Table 5: Risk of incident T2DM in men according to serum testosterone category at baseline.**

| **MEN**  **(N= 70,541)** |  | **aIRR (95% CI)** |  |
| --- | --- | --- | --- |
|  | Adjusted (Model 1) | Adjusted (Model 2) | Adjusted (Model 3) |
| **Serum testosterone concentration categories (nmol/L)** |  |  |  |
| ***< 7*** | 3.82 (3.31-4.41); p<0.001 | 2.60 (2.25-3.00); p<0.001 | 2.71 (2.34-3.14); p<0.001 |
| ***7 - 9.99*** | 3.70 (3.24-4.22); p<0.001 | 2.46 (2.15-2.81); p<0.001 | 2.57 (2.24-2.94); p<0.001 |
| ***10 - 14.99*** | 2.40 (2.13-2.71); p<0.001 | 1.83 (1.62-2.06); p<0.001 | 1.90 (1.68-2.15); p<0.001 |
| ***15 - 19.99*** | 1.45 (1.27-1.66); p<0.001 | 1.25 (1.09-1.43); p=0.001 | 1.29 (1.13-1.47); p<0.001 |
| ***> 20*** | Ref | Ref | Ref |
| **Age** | 1.02 (1.02-1.03); p<0.001 | 1.03 (1.02-1.03); p<0.001 | 1.03 (1.03-1.03); p<0.001 |
| **BMI (kg/m^2^) categorised** | | | |
| **<25** |  | Ref | Ref |
| **25-30** |  | 2.06 (1.82-2.33); p<0.001 | 2.11 (1.86-2.38); p<0.001 |
| **>30** |  | 5.47 (4.85-6.17); p<0.001 | 5.48 (4.85-6.18); p<0.001 |
| ***Missing or implausible data*** |  | 1.68 (1.43-1.96); p<0.001 | 1.72 (1.46-2.02); p<0.001 |
| ***Townsend index*** |  |  |  |
| ***1 (least deprived)*** |  |  | Ref |
| **2** |  |  | 1.09 (0.99-1.21); p=0.087 |
| **3** |  |  | 1.30 (1.17-1.44); p<0.001 |
| **4** |  |  | 1.40 (1.25-1.56); p<0.001 |
| **5 (most deprived)** |  |  | 1.59 (1.40-1.80); p<0.001 |
| ***Missing or implausible data*** |  |  | 1.31 (1.06-1.61); p=0.012 |
| **Smoking status** | | | |
| **Non-smokers** |  |  | Ref |
| **Smokers** |  |  | 1.38 (1.26-1.50); p<0.001 |
| **Missing or implausible data** |  |  | 1.04 (0.82-1.32); p=0.742 |

*Model 1 – Adjusted for age*

*Model 2 – Adjusted for age and BMI*

*Model 3 – Adjusted for age, BMI, townsend index and smoking status*

**Supplementary Table 6: Risk of incident T2DM in men according to the serum SHBG category at baseline.**

| **MEN**  **(N= 15,907)** |  | **aIRR (95% CI); p-value** |  |
| --- | --- | --- | --- |
|  | Adjusted (Model 1) | Adjusted (Model 2) | Adjusted (Model 3) |
| **Serum SHBG concentration categories (nmol/L)** |  |  |  |
| ***<20*** | 8.23 (5.37-12.63); p<0.001 | 5.00 (3.24-7.71); p<0.001 | 5.74 (3.72-8.87); p<0.001 |
| ***20 - 29.99*** | 4.30 (2.83-6.53); p<0.001 | 2.92 (1.91-4.44); p<0.001 | 3.20 (2.09-4.87); p<0.001 |
| ***30 - 39.99*** | 3.33 (2.19-5.08); p<0.001 | 2.45 (1.60-3.74); p<0.001 | 2.61 (1.71-3.99); p<0.001 |
| ***40 - 49.99*** | 1.56 (0.98-2.50); p=0.063 | 1.28 (0.80-2.06); p=0.298 | 1.36 (0.85-2.17); p=0.207 |
| ***50 - 59.99*** | 1.07 (0.61-1.87); p=0.825 | 0.88 (0.50-1.54); p=0.654 | 0.91 (0.52-1.60); p=0.748 |
| ***≥60*** | Ref | Ref | Ref |
| **Age** | 1.04 (1.04-1.05); p<0.001 | 1.04 (1.04-1.05); p<0.001 | 1.05 (1.04-1.05); p<0.001 |
| **BMI (kg/m^2^) categorised** |  |  |  |
| **<25** |  | Ref | Ref |
| **25-30** |  | 1.90 (1.41-2.55); p<0.001 | 1.96 (1.46-2.64); p<0.001 |
| **>30** |  | 5.61 (4.23-7.43); p<0.001 | 5.66 (4.26-7.51); p<0.001 |
| ***Missing or implausible data*** |  | 1.95 (1.34-2.82); p<0.001 | 2.04 (1.40-2.98); p<0.001 |
| ***Townsend index*** |  |  |  |
| ***1 (least deprived)*** |  |  | Ref |
| **2** |  |  | 1.23 (0.98-1.55); p=0.073 |
| **3** |  |  | 1.44 (1.14-1.82); p=0.002 |
| **4** |  |  | 1.73 (1.36-2.19); p<0.001 |
| **5 (most deprived)** |  |  | 1.75 (1.35-2.25); p<0.001 |
| ***Missing or implausible data*** |  |  | 1.22 (0.76-1.94); p=0.416 |
| **Smoking status** |  |  |  |
| **Non-smokers** |  |  | Ref |
| **Smokers** |  |  | 1.57 (1.31-1.89); p<0.001 |
| **Missing or implausible data** |  |  | 0.90 (0.47-1.74); p=0.764 |
|  |  |  |  |

*Model 1 – Adjusted for age*

*Model 2 – Adjusted for age and BMI*

*Model 3 – Adjusted for age, BMI, townsend index and smoking status*

**Supplementary Table 7: Risk of incident T2DM in women according to the serum category testosterone at baseline.**

| **WOMEN**  **(N=81,889)** | **aIRR (95% CI); p-value** | | | |
| --- | --- | --- | --- | --- |
|  | Adjusted (Model 1) | Adjusted (Model 2) | Adjusted (Model 3) | Adjusted (Model 4) |
| **Serum testosterone concentration categories (nmol/L)** |  |  |  |  |
| ***< 1*** | Ref | Ref | Ref | Ref |
| ***1 - 1.49*** | 1.21 (1.02-1.43); p=0.030 | 1.12 (0.95-1.33); p=0.184 | 1.12 (0.94-1.32); p=0.204 | 1.11 (0.94-1.32); p=0.213 |
| ***1.5 - 1.99*** | 1.45 (1.23-1.70); p<0.001 | 1.26 (1.07-1.48); p=0.005 | 1.23 (1.05-1.45); p=0.011 | 1.23 (1.04-1.44); p=0.013 |
| ***2 - 2.49*** | 1.70 (1.42-2.04); p<0.001 | 1.34 (1.12-1.61); p=0.002 | 1.30 (1.08-1.56); p=0.005 | 1.28 (1.07-1.54); p=0.008 |
| ***2.5 - 2.99*** | 2.07 (1.67-2.58); p<0.001 | 1.59 (1.27-1.97); p<0.001 | 1.53 (1.23-1.90); p<0.001 | 1.50 (1.20-1.87); p<0.001 |
| ***3 - 3.49*** | 2.51 (1.90-3.32); p<0.001 | 1.74 (1.31-2.30); p<0.001 | 1.68 (1.27-2.23); p<0.001 | 1.62 (1.22-2.15); p<0.001 |
| ***≥ 3.5*** | 3.00 (2.36-3.82); p<0.001 | 2.09 (1.64-2.67); p<0.001 | 1.98 (1.55-2.52); p<0.001 | 1.89 (1.48-2.42); p<0.001 |
| **Age** | 1.05 (1.05-1.06); p<0.001 | 1.05 (1.05-1.06); p<0.001 | 1.05 (1.05-1.06); p<0.001 | 1.05 (1.05-1.06); p<0.001 |
| **BMI (kg/m^2^) categorised** |  |  |  |  |
| ***<25*** |  | Ref | Ref | Ref |
| ***25-30*** |  | 3.80 (2.96-4.87); p<0.001 | 3.72 (2.90-4.77); p<0.001 | 3.69 (2.88-4.74); p<0.001 |
| ***>30*** |  | 15.29 (12.30-19.02); p<0.001 | 14.58 (11.72-18.14); p<0.001 | 14.39 (11.57-17.91); p<0.001 |
| ***Missing or implausible data*** |  | 4.19 (3.19-5.51); p<0.001 | 3.93 (2.96-5.22); p<0.001 | 3.93 (2.96-5.21); p<0.001 |
| ***Townsend index*** |  |  |  |  |
| **1 (least deprived)** |  |  | Ref | Ref |
| **2** |  |  | 1.21 (1.00-1.45); p=0.045 | 1.20 (1.00-1.45); p=0.049 |
| ***3*** |  |  | 1.40 (1.17-1.67); p<0.001 | 1.40 (1.17-1.67); p<0.001 |
| ***4*** |  |  | 1.67 (1.40-1.99); p<0.001 | 1.66 (1.40-1.98); p<0.001 |
| ***5 (most deprived)*** |  |  | 1.76 (1.45-2.13); p<0.001 | 1.75 (1.45-2.12); p<0.001 |
| ***Missing or implausible data*** |  |  | 1.17 (0.84-1.62); p=0.350 | 1.17 (0.84-1.62); p=0.346 |
| **Smoking status** |  |  |  |  |
| **Non-smokers** |  |  | Ref | Ref |
| **Smokers** |  |  | 1.26 (1.10-1.43); p<0.001 | 1.26 (1.11-1.44); p<0.001 |
| **Missing or implausible data** |  |  | 1.37 (1.00-1.88); p=0.047 | 1.37 (1.00-1.88); p=0.047 |
| **PCOS** |  |  |  | 1.35 (1.10-1.65); p<0.001 |
| **PCOS/Anovulation/Hirsutism** |  |  |  |  |

*Model 1 – Adjusted for age*

*Model 2 – Adjusted for age and BMI*

*Model 3 – Adjusted for age, BMI, townsend index and smoking status*

*Model 4 – Adjusted for age, BMI, townsend index, smoking status and PCOS/*

*Model 5 – Adjusted for age, BMI, townsend index, smoking status and PCOS/Anovulation/Hirsutism*

**Supplementary Table 8: Risk of incident T2DM in women according to the serum SHBG category at baseline**

| **WOMEN**  **(N=42,034)** | **aIRR (95% CI); p-value** | | | |
| --- | --- | --- | --- | --- |
|  | Adjusted (Model 1) | Adjusted (Model 2) | Adjusted (Model 3) | Adjusted (Model 4) |
| **Serum SHBG concentration categories (nmol/L)** |  |  |  |  |
| ***<20*** | 19.76 (14.36-27.21); p<0.001 | 8.96 (6.42-12.50); p<0.001 | 9.23 (6.61-12.88); p<0.001 | 9.13 (6.53-12.75); p<0.001 |
| ***20 - 29.99*** | 8.66 (6.29-11.93) ; p<0.001 | 4.45 (3.20-6.19) ; p<0.001 | 4.48 (3.22-6.24) ; p<0.001 | 4.44 (3.19-6.18) ; p<0.001 |
| ***30 - 39.99*** | 4.66 (3.31-6.57) ; p<0.001 | 2.69 (1.90-3.82) ; p<0.001 | 2.70 (1.91-3.84) ; p<0.001 | 2.69 (1.90-3.82) ; p<0.001 |
| ***40 - 49.99*** | 2.99 (2.04-4.38) ; p<0.001 | 2.05 (1.40-3.02) ; p<0.001 | 2.08 (1.41-3.05) ; p<0.001 | 2.07 (1.41-3.05) ; p<0.001 |
| ***50 - 59.99*** | 1.64 (1.02-2.64) ; p=0.043 | 1.29 (0.80-2.08) ; p=0.295 | 1.29 (0.80-2.07) ; p=0.304 | 1.29 (0.80-2.08) ; p=0.301 |
| ***≥60*** | Ref | Ref | Ref | Ref |
| ***Age*** | 1.06 (1.05-1.06) ; p<0.001 | 1.06 (1.05-1.07) ; p<0.001 | 1.06 (1.06-1.07) ; p<0.001 | 1.06 (1.06-1.07) ; p<0.001 |
| **BMI (kg/m^2^) categorised** |  |  |  |  |
| ***<25*** |  | Ref | Ref | Ref |
| ***25-30*** |  | 2.87 (1.99-4.13) ; p<0.001 | 2.82 (1.95-4.06) ; p<0.001 | 2.81 (1.95-4.05) ; p<0.001 |
| ***>30*** |  | 7.68 (5.52-10.68) ; p<0.001 | 7.25 (5.21-10.09) ; p<0.001 | 7.21 (5.18-10.03) ; p<0.001 |
| ***Missing or implausible data*** |  | 2.86 (1.89-4.31) ; p<0.001 | 2.79 (1.83-4.26) ; p<0.001 | 2.78 (1.83-4.24) ; p<0.001 |
| ***Townsend index*** |  |  |  |  |
| **1 (least deprived)** |  |  |  |  |
| **2** |  |  | Ref | Ref |
| ***3*** |  |  | 1.16 (0.88-1.54) ; p=0.285 | 1.16 (0.88-1.54) ; p=0.289 |
| ***4*** |  |  | 1.43 (1.10-1.86) ; p=0.008 | 1.43 (1.10-1.86) ; p=0.008 |
| ***5 (most deprived)*** |  |  | 1.52 (1.16-1.99) ; p=0.002 | 1.52 (1.16-1.98) ; p=0.002 |
| ***Missing or implausible data*** |  |  | 1.89 (1.44-2.49) ; p<0.001 | 1.89 (1.44-2.48) ; p<0.001 |
| **Smoking status** |  |  |  |  |
| **Non-smokers** |  |  | Ref | Ref |
| **Smokers** |  |  | 1.26 (1.04-1.53) ; p=0.018 | 1.26 (1.04-1.53) ; p=0.018 |
| **Missing or implausible data** |  |  | 1.11 (0.68-1.82) ; p=0.682 | 1.11 (0.68-1.82) ; p=0.677 |
| **PCOS** |  |  |  | 1.19 (0.91-1.55); p=0.193 |
| **PCOS/Anovulation/Hirsutism** |  |  |  |  |

*Model 1 – Adjusted for age*

*Model 2 – Adjusted for age and BMI*

*Model 3 – Adjusted for age, BMI, townsend index and smoking status*

*Model 4 – Adjusted for age, BMI, townsend index, smoking status and PCOS*

*Model 5 – Adjusted for age, BMI, townsend index, smoking status and PCOS/Anovulation/Hirsutism*

**Suppl. Table 9: Risk of incident T2DM and serum testosterone concentration in men <50 years of age (N=29,857).**

| **MEN < 50 years old** | **IRR (95% CI); p-value** | | |
| --- | --- | --- | --- |
|  | **Adjusted^1^** | **Adjusted^2^** | **Adjusted3** |
| **Serum testosterone concentration categories (nmol/L)** |  |  |  |
| < 7 | 4.90 (3.72-6.44) ; p<0.001 | 3.01 (2.27-3.98) ; p<0.001 | 3.10 (2.34-4.10) ; p<0.001 |
| 7 - 9.99 | 4.25 (3.31-5.45) ; p<0.001 | 2.62 (2.03-3.39) ; p<0.001 | 2.76 (2.13-3.56) ; p<0.001 |
| 10 - 14.99 | 2.36 (1.88-2.97) ; p<0.001 | 1.71 (1.35-2.16) ; p<0.001 | 1.80 (1.42-2.28) ; p<0.001 |
| 15 - 19.99 | 1.28 (0.99-1.65) ; p=0.063 | 1.09 (0.84-1.42) ; p=0.497 | 1.14 (0.88-1.48) ; p=0.317 |
| > 20 | Ref | Ref | Ref |

^1^ *Adjusted for age,*

^2^ *Adjusted for age, BMI,*

^3^ *Adjusted for age, BMI, Townsend index, smoking status*

**Suppl.Table 10: Risk of incident T2DM and serum testosterone concentration in men ≥50 years of age (N=40,684).**

| **MEN ≥ 50 years old** | **IRR (95% CI); p-value** | | |
| --- | --- | --- | --- |
|  | **Adjusted^1^** | **Adjusted^2^** | **Adjusted^3^** |
| **Serum testosterone concentration categories (nmol/L)** |  |  |  |
| < 7 | 3.87 (3.27-4.59) ; p<0.001 | 2.69 (2.27-3.19) ; p<0.001 | 2.82 (2.38-3.35) ; p<0.001 |
| 7 - 9.99 | 3.44 (2.94-4.01) ; p<0.001 | 2.40 (2.05-2.80) ; p<0.001 | 2.50 (2.13-2.93) ; p<0.001 |
| 10 - 14.99 | 2.34 (2.03-2.69) ; p<0.001 | 1.84 (1.60-2.13) ; p<0.001 | 1.92 (1.66-2.21) ; p<0.001 |
| 15 - 19.99 | 1.47 (1.26-1.72) ; p<0.001 | 1.28 (1.10-1.50) ; p=0.002 | 1.32 (1.13-1.55) ; p<0.001 |
| > 20 | Ref | Ref | Ref |

^1^ *Adjusted for age,*

^2^ *Adjusted for age, BMI,*

^3^ *Adjusted for age, BMI, Townsend index, smoking status*

**Suppl.Table 11: Risk of incident T2DM and serum testosterone concentration in women <50 years of age (N=76,224)**

| **WOMEN < 50 years old** | **IRR (95% CI); p-value** | | | | | | |
| --- | --- | --- | --- | --- | --- | --- | --- |
|  | **Adjusted^1^** | **Adjusted^2^** | | **Adjusted^3^** | | **Adjusted^4^** | |
| **Serum testosterone concentration categories (nmol/L)** |  | |  | |  | |  |
| < 1 | Ref | | Ref | | Ref | | Ref |
| 1.0 - 1.49 | 1.14 (0.94-1.38) ; p=0.177 | | 1.01 (0.84-1.22) ; p=0.893 | | 1.01 (0.84-1.23) ; p=0.883 | | 1.01 (0.83-1.22) ; p=0.927 |
| 1.5 - 1.99 | 1.38 (1.16-1.65) ; p<0.001 | | 1.16 (0.97-1.38) ; p=0.108 | | 1.15 (0.96-1.38) ; p=0.129 | | 1.14 (0.95-1.36) ; p=0.156 |
| 2.0 - 2.49 | 1.59 (1.31-1.95) ; p<0.001 | | 1.20 (0.98-1.47) ; p=0.074 | | 1.18 (0.96-1.44) ; p=0.109 | | 1.16 (0.94-1.41) ; p=0.160 |
| 2.5 - 2.99 | 1.88 (1.47-2.40) ; p<0.001 | | 1.33 (1.04-1.70) ; p=0.024 | | 1.30 (1.01-1.66) ; p=0.041 | | 1.26 (0.98-1.61) ; p=0.072 |
| 3.0 - 3.49 | 2.54 (1.87-3.44) ; p<0.001 | | 1.61 (1.19-2.19) ; p=0.002 | | 1.58 (1.16-2.14) ; p=0.003 | | 1.50 (1.10-2.04) ; p=0.010 |
| ≥ 3.5 | 3.07 (2.30-4.10) ; p<0.001 | | 1.93 (1.44-2.58) ; p<0.001 | | 1.84 (1.37-2.46) ; p<0.001 | | 1.73 (1.29-2.32) ; p<0.001 |

^1^ *Adjusted for age,*

^2^ *Adjusted for age, BMI,*

^3^ *Adjusted for age, BMI, Townsend index, smoking status*

^4^ *Adjusted for age, BMI, Townsend index, smoking status, PCOS*

**Suppl. Table 12: Risk of incident T2DM and serum testosterone concentration in a cohort of women ≥50 years of age (N=5,665)**

| **WOMEN ≥ 50 years old** | **IRR (95% CI); p-value** | | | |
| --- | --- | --- | --- | --- |
|  | **Adjusted^1^** | **Adjusted^2^** | **Adjusted^3^** | **Adjusted^4^** |
| **Serum testosterone concentration categories (nmol/L)** |  |  |  |  |
| < 1 | Ref | Ref | Ref | Ref |
| 1.0 - 1.49 | 1.41 (0.98-2.04) ; p=0.067 | 1.44 (1.00-2.09) ; p=0.052 | 1.44 (1.00-2.09) ; p=0.053 | 1.44 (0.99-2.08) ; p=0.053 |
| 1.5 - 1.99 | 1.70 (1.16-2.50) ; p=0.006 | 1.60 (1.09-2.34) ; p=0.016 | 1.54 (1.05-2.25) ; p=0.028 | 1.54 (1.05-2.25) ; p=0.028 |
| 2.0 - 2.49 | 2.73 (1.77-4.22) ; p<0.001 | 2.28 (1.48-3.53) ; p<0.001 | 2.13 (1.37-3.30) ; p<0.001 | 2.12 (1.37-3.29) ; p<0.001 |
| 2.5 - 2.99 | 4.90 (3.09-7.78) ; p<0.001 | 4.24 (2.67-6.74) ; p<0.001 | 4.17 (2.61-6.66) ; p<0.001 | 4.15 (2.60-6.63) ; p<0.001 |
| 3.0 - 3.49 | 3.71 (1.80-7.68) ; p<0.001 | 2.88 (1.39-5.95) ; p=0.004 | 2.81 (1.35-5.82) ; p=0.006 | 2.82 (1.36-5.84) ; p=0.005 |
| ≥ 3.5 | 4.72 (2.99-7.45) ; p<0.001 | 3.88 (2.45-6.13) ; p<0.001 | 3.73 (2.35-5.90) ; p<0.001 | 3.71 (2.34-5.87) ; p<0.001 |

^1^ *Adjusted for age,*

^2^ *Adjusted for age, BMI,*

^3^ *Adjusted for age, BMI, Townsend index, smoking status*

^4^ *Adjusted for age, BMI, Townsend index, smoking status, PCOS*

**Suppl. Table 13: Risk of incident T2DM and serum SHBG concentration in men <50 years of age (N=6,710).**

| **MEN < 50 years old** | **IRR (95% CI); p-value** | | |  |
| --- | --- | --- | --- | --- |
|  | **Adjusted^1^** | **Adjusted^2^** | **Adjusted^3^** | |
| **Serum SHBG concentration categories (nmol/L)** |  |  |  | |
| < 20 | 7.55 (1.86-30.70) ; p=0.005 | 4.37 (1.07-17.87) ; p=0.040 | 4.99 (1.21-20.50) ; p=0.026 | |
| 20 – 29.99 | 4.85 (1.19-19.79) ; p=0.028 | 3.28 (0.80-13.43) ; p=0.099 | 3.53 (0.86-14.53) ; p=0.080 | |
| 30 – 39.99 | 2.13 (0.50-9.05) ; p=0.307 | 1.67 (0.39-7.10) ; p=0.490 | 1.74 (0.41-7.43) ; p=0.453 | |
| 40 – 49.99 | 2.25 (0.50-10.07) ; p=0.287 | 2.04 (0.46-9.11) ; p=0.352 | 2.08 (0.47-9.34) ; p=0.337 | |
| 50 – 59.99 | 1.43 (0.26-7.79) ; p=0.682 | 1.34 (0.25-7.31) ; p=0.736 | 1.40 (0.26-7.65) ; p=0.698 | |
| ≥ 60 | Ref | Ref | Ref | |

^1^ *Adjusted for age,*

^2^ *Adjusted for age, BMI,*

^3^ *Adjusted for age, BMI, Townsend index, smoking status*

**Suppl. Table 14: Risk of incident T2DM and serum SHBG concentration in men ≥50 years of age (N=9,197).**

| **MEN ≥ 50 years old** | **IRR (95% CI); p-value** | | |  |
| --- | --- | --- | --- | --- |
|  | **Adjusted^1^** | **Adjusted^2^** | **Adjusted^3^** | |
| **Serum SHBG concentration categories (nmol/L)** |  |  |  | |
| < 20 | 7.60 (4.81-12.01) ; p<0.001 | 5.09 (3.20-8.08) ; p<0.001 | 5.93 (3.72-9.44) ; p<0.001 | |
| 20 – 29.99 | 3.64 (2.34-5.66) ; p<0.001 | 2.64 (1.69-4.12) ; p<0.001 | 2.92 (1.87-4.57) ; p<0.001 | |
| 30 – 39.99 | 3.27 (2.11-5.09) ; p<0.001 | 2.53 (1.62-3.94) ; p<0.001 | 2.73 (1.75-4.27) ; p<0.001 | |
| 40 – 49.99 | 1.35 (0.82-2.23) ; p=0.243 | 1.15 (0.69-1.91) ; p=0.585 | 1.23 (0.74-2.04) ; p=0.419 | |
| 50 – 59.99 | 0.98 (0.54-1.78) ; p=0.941 | 0.82 (0.45-1.50) ; p=0.528 | 0.86 (0.47-1.57) ; p=0.628 | |
| ≥ 60 | Ref | Ref | Ref | |

^1^ *Adjusted for age,*

^2^ *Adjusted for age, BMI,*

^3^ *Adjusted for age, BMI, Townsend index, smoking status*

**Suppl. Table 15: Risk of incident T2DM and serum SHBG concentration in women <50 years of age (N=39,713).**

| **WOMEN < 50 years old** | **IRR (95% CI); p-value** | | | |
| --- | --- | --- | --- | --- |
|  | **Adjusted^1^** | **Adjusted^2^** | **Adjusted^3^** | **Adjusted^4^** |
| **Serum SHBG concentration categories (nmol/L)** |  |  |  |  |
| < 20 | 19.76 (14.36-27.21) ; p<0.001 | 9.53 (6.50-13.97) ; p<0.001 | 9.72 (6.63-14.25) ; p<0.001 | 9.57 (6.52-14.05) ; p<0.001 |
| 20 – 29.99 | 8.66 (6.29-11.93) ; p<0.001 | 4.66 (3.18-6.83) ; p<0.001 | 4.63 (3.16-6.80) ; p<0.001 | 4.57 (3.11-6.71) ; p<0.001 |
| 30 – 39.99 | 4.66 (3.31-6.57) ; p<0.001 | 2.78 (1.86-4.17) ; p<0.001 | 2.81 (1.88-4.21) ; p<0.001 | 2.80 (1.87-4.19) ; p<0.001 |
| 40 – 49.99 | 2.99 (2.04-4.38) ; p<0.001 | 1.81 (1.15-2.86) ; p=0.011 | 1.81 (1.14-2.86) ; p=0.011 | 1.80 (1.14-2.85) ; p=0.012 |
| 50 – 59.99 | 1.64 (1.02-2.64) ; p=0.043 | 1.16 (0.66-2.05) ; p=0.613 | 1.16 (0.66-2.06) ; p=0.605 | 1.16 (0.66-2.06) ; p=0.604 |
| ≥ 60 | Ref | Ref | Ref | Ref |

^1^ *Adjusted for age,*

^2^ *Adjusted for age, BMI,*

^3^ *Adjusted for age, BMI, Townsend index, smoking status*

^4^ *Adjusted for age, BMI, Townsend index, smoking status, PCOS*

**Suppl. Table 16: Risk of incident T2DM and serum SHBG concentration in women ≥50 years of age (N=2,321).**

| **WOMEN ≥ 50 years old** | **IRR (95% CI); p-value** | | | |
| --- | --- | --- | --- | --- |
|  | **Adjusted^1^** | **Adjusted^2^** | **Adjusted^3^** | **Adjusted^4^** |
| **Serum SHBG concentration categories (nmol/L)** |  |  |  |  |
| < 20 | 12.65 (6.39-25.02) ; p<0.001 | 7.73 (3.81-15.68) ; p<0.001 | 8.18 (3.98-16.80) ; p<0.001 | 8.40 (4.09-17.27) ; p<0.001 |
| 20 – 30 | 6.05 (3.15-11.59) ; p<0.001 | 4.05 (2.08-7.89) ; p<0.001 | 4.34 (2.22-8.46) ; p<0.001 | 4.31 (2.21-8.41) ; p<0.001 |
| 30 – 40 | 3.67 (1.81-7.43) ; p<0.001 | 2.51 (1.22-5.15) ; p=0.012 | 2.53 (1.23-5.21) ; p=0.011 | 2.46 (1.19-5.06) ; p=0.015 |
| 40 – 50 | 3.94 (1.94-7.98) ; p<0.001 | 2.85 (1.39-5.82) ; p=0.004 | 2.91 (1.42-5.95) ; p=0.004 | 2.96 (1.45-6.06) ; p=0.003 |
| 50 – 60 | 2.00 (0.83-4.84) ; p=0.122 | 1.70 (0.70-4.11) ; p=0.240 | 1.59 (0.66-3.86) ; p=0.304 | 1.62 (0.67-3.94) ; p=0.285 |
| ≥ 60 | Ref | Ref | Ref | Ref |

^1^ *Adjusted for age,*

^2^ *Adjusted for age, BMI,*

^3^ *Adjusted for age, BMI, Townsend index, smoking status*

^4^ *Adjusted for age, BMI, Townsend index, smoking status, PCOS*

**Supplementary Figure 1: Risk of incident diabetes in men (N=70,541) and women (N=81,889) of the testosterone cohort stratified by age.**

**
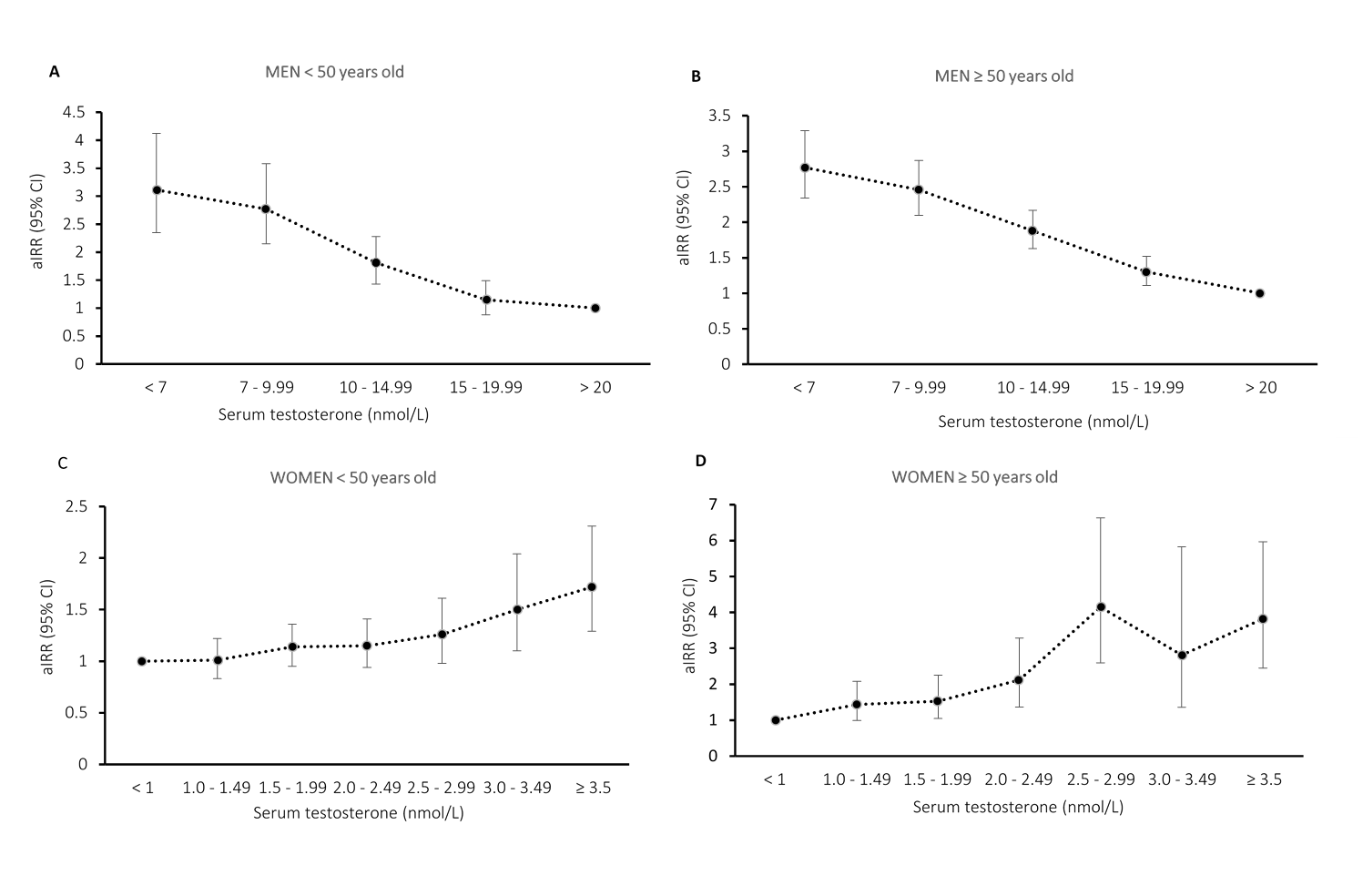
**

**Supplementary Figure 2: Risk of incident diabetes in men (N=15,907) and in women (N=42,034) of the SHBG cohort stratified by age.**

**
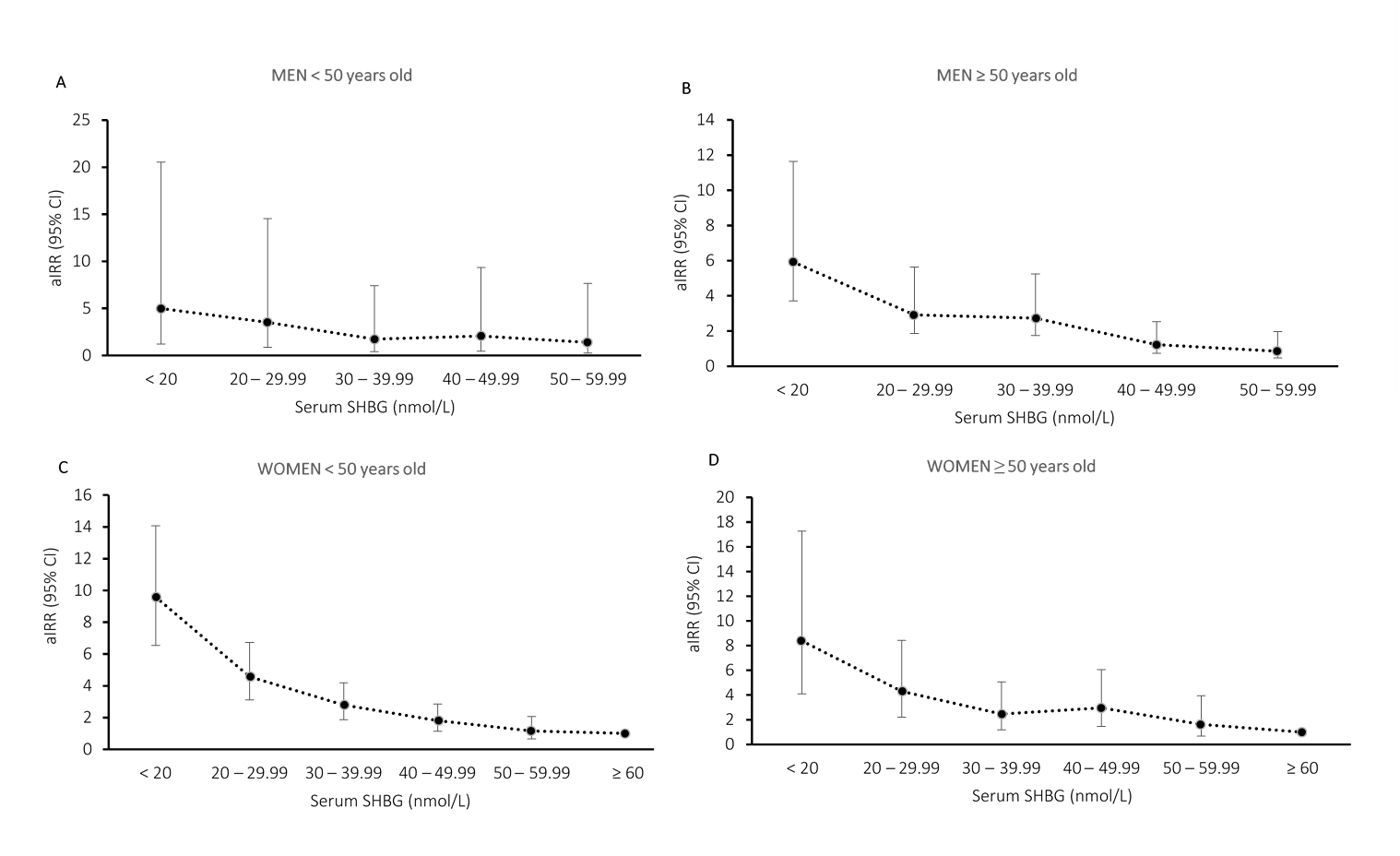
**
